# The B cell receptor signaling pathway in mantle cell lymphoma

**DOI:** 10.18632/oncotarget.25011

**Published:** 2018-03-27

**Authors:** Maria I. Merolle, Makhdum Ahmed, Krystle Nomie, Michael L. Wang

**Affiliations:** ^1^ Department of Lymphoma and Myeloma, MD Anderson Cancer Center, Houston, TX, USA

**Keywords:** B cell receptor, mantle cell lymphoma, B cell signaling pathway, targeted therapy

## Abstract

Signal transduction through the constitutively activated B cell receptor (BCR) plays a key role in the pathogenesis of B-cell tumors by promoting survival and proliferation of malignant B cells. The BCR signaling pathway is known to be deregulated in Mantle Cell Lymphoma (MCL) due to mutations or epigenetic events that impact regulatory proteins. One such protein is Bruton's tyrosine kinase (BTK), an integral component of the BCR signaling pathway. The success of ibrutinib, a BTK inhibitor, and other drugs that target components of the BCR pathway is evidence that regulation of the BCR signaling pathway is an effective method of MCL treatment. The complexity of the pathway indicates that it contains other potential therapeutic targets for the treatment of MCL. This is supported by recent and ongoing clinical trials of inhibitors of molecules such as PI3K, BCL-2, and BTK that show promising initial results. Additionally, agents that target different points of the pathway may have synergistic effects when used in combination. This review provides a description of the BCR signaling pathway on the molecular level followed by an explanation of its relationship to MCL. The role of the BCR signaling pathway in the pathogenesis of MCL is explained through an overview of the drugs that target BCR signaling in MCL treatment.

## INTRODUCTION

**Figure 1:**
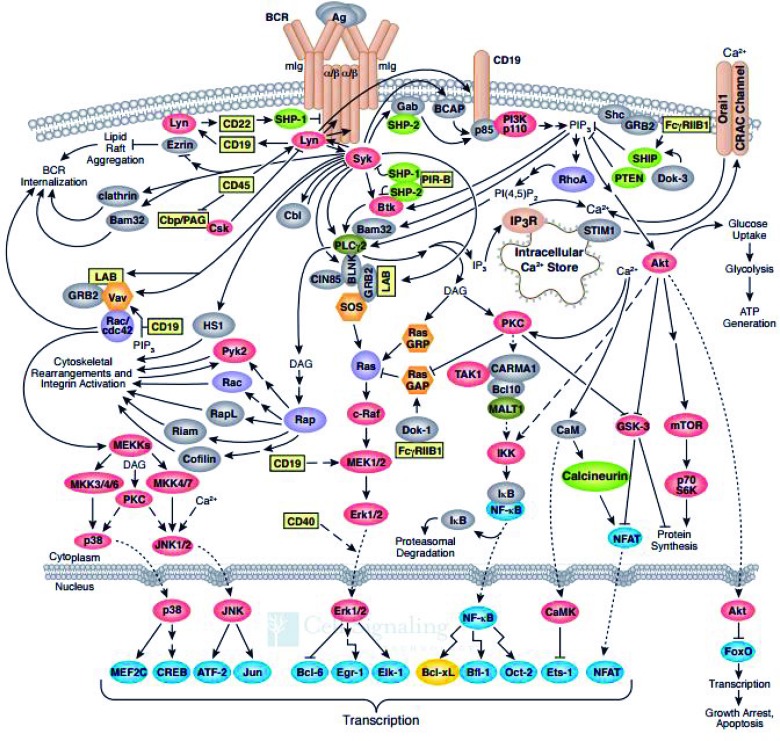
B-cell receptor signaling pathway

B cells mediate the humoral immune response, one component of the activity of the adaptive immune system. B cells have several receptors that transduce external signals and influence the fate of the B cell. However, the principal signaling pathway in B cell activity is the B Cell Antigen-Receptor (BCR) pathway, shown in Figure 1, reproduced with permission by Cell Signaling Technology®. There are several possible responses that may be signaled in a B cell, such as apoptosis, proliferation, and differentiation into memory B cells or B cells that produce antibodies [1]. Each B cell has receptors that are specific to a certain antigen. If a B cell never binds with its antigen, it will eventually undergo apoptosis. Upon antigenation of the B cell, the immune response will be triggered and the B cell will differentiate and proliferate. In MCL, B cells undergo constitutive proliferation due to deregulation of the BCR pathway.

## SIGNAL INITIATION

### Structure of the BCR complex

The B-cell Receptor Complex is composed of a membrane immunoglobulin (IgM) disulfide-bonded to the heterodimers CD79a and CD79b [1]. IgM is composed of 2 immunoglobulin heavy chains and 2 light chains. The cytoplasmic regions of CD79a and CD79b are called ITAMs (immunoreceptor tyrosine-based activation motif). When the specific antigen binds to the receptor's active site, the ITAM is phosphorylated by Lyn, an Src family kinase, and is then able to bind to the SH2 domains of Syk, a tyrosine kinase [2].

### Formation of the signalosome and B cell activation

Syk is activated when it binds to the phosphorylated ITAM, and it subsequently activates proteins that are recruited to the BCR complex by Lyn. Lyn's activity recruits protein tyrosine kinases and adaptor proteins to form the signalosome, a group of proteins that aggregate with Lyn and Syk upon BCR ligation [1]. This complex includes Bruton's tyrosine kinase (BTK), Grb2, and B Cell Linker (BLNK). More detail on the formation of this complex will be given in the next section. Other membrane proteins, such as CD19, complex with Lyn and assist in B cell activation by lowering the receptor's threshold for antigen stimulation [3]. Indeed, members of many clusters of differentiation (CD) act as such co-stimulatory molecules, including CD19, CD20, CD21, CD23, and CD45 [4]. BTK is activated by Syk and sends a signal that stimulates actin polymerization at the cell cortex, leading to a reconfiguration of the actin cytoskeleton [5]. An inhibitory factor, SH2-containing inositol-5 phosphatase-1 (SHIP-1), removes actin from the cortex to promote cell contraction. SHIP-1 has also been shown to be necessary for efficient actin remodeling, balancing the stimulatory effect of BTK. Dynamic actin remodeling modulated by BTK and SHIP-1 causes B cell receptors to aggregate in lipid rafts on the cell membrane, which attenuates the BCR signal [5].

## SIGNAL PROPAGATION

The B cell receptor ligation leads to a complex of several proteins (the signalosome), which activate several downstream pathways that propagate the B cell response signal. The principle components in this signal propagation are described below.

### Bruton's tyrosine kinase

BLNK is associated with CD79a and then is phosphorylated by Syk. BLNK recruits BTK via its Src Homology 2 (SH2) domain, and then BTK is also phosphorylated by Syk [6]. BTK plays a critical role in amplifying the B cell activation signal. The importance of BTK is evidenced by the fact that a BTK loss-of-function mutation is responsible for X-linked agammaglobulinemia (XLA), which is characterized by deficiency of mature B-cells and antibody production [7]. BTK's function is largely dependent on its pleckstrin homology domain, which allows it to be recruited to the membrane by phosphatidylinositol (3,4,5)-trisphosphate (PIP_3_) to facilitate the PI3K/Akt pathway reactions [1]. Btk activation is amplified through autophosphorylation of the Y223 site in its Src Homology 3 (SH3) domain [8]. Btk has been shown to activate IκB kinase, which phosphorylates the NF-κB inhibitor I-κBα, allowing NF-κB to translocate to the nucleus [9]. BTK's most consequential role in the BCR pathway is its activation of phospholipase C-γ2 (PLC-γ2), which is largely responsible for triggering subsequent pathways that yield important transcription factors. Important effectors activated in the pathways downstream of PLC-γ2 include NF-κB, which is a product of multiple pathways within the B-cell, and nuclear factor of activated T cells (NFAT) [7].

### Phospholipase C-γ2

BTK and Syk phosphorylate phospholipase C-γ2 (PLC-γ2), which hydrolyzes phosphatidylinositol 4,5-biphosphate (PIP_2_) to form diaglycerol (DAG) and inositol triphosphate (IP_3_) [10]. IP_3_ is involved in an increase in intracellular calcium concentration, via release of Ca^2+^ from the endoplasmic reticulum and influx of Ca^2+^ from outside of the cell [11]. Cytosolic Ca^2+^ facilitates the activation of calcineurin by calcimodulin, which directly activates NFAT. NFAT regulates the expression of many genes [7]. Ca^2+^ also works in combination with DAG to activate the β isoform of protein kinase-C (PKC) [12]. The vital activity of PLC-γ2 underscores the significance of BTK, without which the important substrates NF-κB and NFAT would not be activated.

PLCγ2 is also involved in the activation of mitogen-activated protein kinase (MAPK) pathways, including the extracellular signal-regulated kinase 1/2 (ERK1/2), *c-jun* NH2-terminal kinase (JNK) and p38 MAPK [1]. Eventually, ERK kinase activity leads to down-regulation of pro-apoptotic activity of BCL-2-interacting mediator of cell death (BIM) protein [13]. Apart from being activated by PLC-γ2 signaling, MAPK pathway can be also turned on by RAS oncoprotein, although a signal from PLC-γ2 is also implicated in the activation of RAS. This PLC-γ2 signal is transduced to RAS through Vav, a guanine nucleotide exchange factor, and growth factor receptor-bound protein 2 (Grb-2), which complexes with the Sos protein. These proteins are recruited by BLNK to the signalosome area when the ITAM domains of CD79a/b are phosphorylated after BCR ligation [13], [2]. It has been observed that after BCR clustering, RAS associates with the aggregate we call the signalosome. RAS is subsequently activated—meaning it is now in the form of RAS-GTP. RAS-GTP binds to the serine/threonine kinases B-RAF and C-RAF. Stimulated RAF kinases phosphorylate and activate MEK1/2, which results in the phosphorylation ERK1/2. Phosphorylated ERK1/2 kinases form dimers, which can then be translocated into the nucleus. ERK1 and ERK2 initiate transcription of regulatory genes such as *c-fos* and *c-jun* that are important for cell survival [14].

### Protein kinase C (PKC)

PKC is a principle effector of the portion of BCR signaling that activates key transcription factor NF-κB [15]. The beta isoform of PKC phosphorylates a caspase recruitment domain-containing protein (CARMA1), which recruits BCL-10 and MALT-1 into what is called the CBM complex [16]. This complex activates IκB kinase, which subsequently phosphorylates IκB, causing its degradation. IκB is initially bound to NF-κB, keeping it inactive. The dissociation of IκB allows NF-κB to be translocated to the nucleus, where it transcribes genes associated with cell proliferation and survival [17]. PKC also affects the chain of events involving RAS that lead to ERK phosphorylation. PKC inhibits RAS-GAP, an inhibitor of RAS, thereby upregulating ERK [18].

### Phosphatidalyinositol-3-kinase

CD19 associates with the BCR during antigen ligation [4]. After BCR ligation, CD19 has been phosphorylated by Lyn, forming a docking site for the SH2 domain of p38, a subunit of phoshphatidalyinositol-3-kinase (PI3K). PI3Ks are lipid kinases that exist in 4 different isoforms: p110α, p110β, p110γ, and p110δ. The p110δ isoform is a key messenger in BCR signaling and is highly expressed in B lymphocytes [19]. PI3K docks via p38 to CD19. This association activates the p110δ subunit of PI3K, allowing PI3K to convert phosphatidylinositol 4,5-biphosphate (PIP_2_) to phosphatidylinositol 3,4,5-triphosphate (PIP_3_) [15]. PIP_3_ at the plasma membrane results in recruitment of Btk and other kinases with pleckstrin homology domains, such as 3-phosphoinositide-dependent protein kinase 1 (PDK-1) and PLC-γ2, amplifying their activity and resulting in continued BCR activation [18]. PDK-1 is also required for the initiation of Akt signaling [13].

PIP_3_ activates Akt by phosphorylating it at the T308 site. Akt is a kinase that phosphorylates and inactivates components required for apoptosis, namely the transcription factors of the FOXO1/3 family and glycogen synthase kinase 3 [1]. Akt also positively regulates Bcl-2 family proteins found on mitochondria that inhibit the mitochondrial intrinsic apoptosis pathway [20]. Akt directly activates the mammalian target of rapamycin (mTOR). The mTOR is a key enzymatic component of two unique protein complexes: mTORC1 and mTORC2 [21]. mTORC1 triggers anabolic pathways necessary for cellular growth and differentiation, such as protein synthesis mediated by the p70-S6 kinase [22]. Thus, Akt can be said to upregulate glycolysis and ATP production, promoting cell survival. mTORC2 functions independently of nutrients and phosphorylates AKT at S473, a required step to achieve maximal AKT activity [21].

### Signal modulation

Every signaling pathway that can result in cell growth contains inhibitory factors meant to prevent the uncontrolled growth that yields malignancies such as MCL. There are several components of the BCR pathway that serve to attenuate the signal. Lyn is one such component, and it functions by phosphorylating tyrosine residues in regulatory proteins [18]. FcγRIIb and CD22 are two of Lyn's targets. FcγRIIb is a transmembrane protein with a cytoplasmic ITIM, which is phosphorylated by Lyn when the FcγRIIb is coligated with the BCR. Active FcγRIIb can recruit SHIP, which dephosphorylates Lyn and Syk to inhibit their activity. SHIP also hydrolyzes PIP_3_, rendering it unable to recruit PLC-γ2, Btk, and Akt to the membrane for signal propagation [1].

CD22 is another inhibitory molecule that can block B cell activation. CD22 contains three ITIMs in its cytoplasmic domain, which are phosphorylated by Lyn upon BCR ligation [32]. Unlike FcγRIIb, CD22 recruits SH2-containing phosphatase 1 (SHP-1), a dephosphorylating enzyme whose targets include Syk, Vav, and BLNK [13].

It is worth giving further description of how CD19 impacts the pathway, as it is an important co-stimulatory protein associated with the BCR. CD19 molecules positively contribute to CD79a/b signaling by enhancing the recruitment and activation of Lyn, PI-3K, Btk, and Vav [15]. CD19 complexes with CD21; when CD21 binds the C3d fragment of a complementary antigen, the complex associates with the BCR that has also been ligated [3]. CD19 can also directly associate with BCR and become activated upon BCR ligation, independent of CD21 [4].

### Tonic vs chronic signaling

It is important to note that the signaling pathway described above represents what is referred to as chronic, or antigen-dependent, BCR signaling. When an antigen ligates the BCR, the signalosome complex is activated, causing cytoskeletal rearrangement that allows for the clustering and communication of multiple BCRs. The BCR clustering sets off the downstream pathways detailed above. However, there is a second type of signal from the BCR, known as tonic signaling [18]. Tonic signaling occurs even when the BCR has not been ligated, and consists mostly of activity dependent on PI3K [13]. It has been shown experimentally that the loss of expression of the BCR complex components in B cells causes cell death [23]. Therefore, tonic BCR signaling is responsible for the basic processes necessary to keep the cell alive and growing.

To review, in chronic BCR signaling, the clustering of B cell receptors with co-stimulatory CD19 recruits PI3K to produce PIP_3,_ which amplifies PLC- γ2 activity. PLC-γ2 activates PKCβ, leading to CARMA-1 activation and CBM complex formation. CBM activity is distinct to chronic BCR signaling [18]. Thus, the main molecular difference between tonic and chronic BCR signaling is the involvement of the CBM complex and subsequent initiation of NF-κB signaling, which is critical in the fate determination of the B cell [16]. However, either form of BCR signaling could be implicated in the development of B cell malignancy.

## RELATED PATHWAYS

A distinct pathway downstream of the B cell receptor that has also been implicated in the pathogenesis of MCL is the NF-κB pathway. The transcription factor NF-κB regulates genes responsible for the cell differentiation and growth that occurs after ligation of the BCR by an antigen. In naïve B cells, NF-κB is found in the cytoplasm. As briefly described in the above section on protein kinase C, inactive NF-κB is bound to the inhibitor IκB, preventing is translocation into the nucleus. The key actor in the activation of NF-κB is the scaffold protein CARMA1, a target of PKC. CARMA1 complexes with MALT1 and Bcl-10, forming the CBM complex [16]. The complex formation of CMB is dependent on BCR signaling because CARMA1 is phosphorylated by PKCβ, which is a downstream product of PLC-γ2 activity.

PI3K is an important factor in BCR signaling and is recruited by CD19 only upon BCR ligation, subsequently producing PIP_3_, which sends continuing recruitment signals to important kinases such as BTK. However, the PI3K/Akt/mTOR pathway is also often viewed as a distinct pathway that is involved in many different processes in B cells. Because the targets of Akt are transcription factors and enzymes involved in protein synthesis, components of the PI3K pathway serve as common therapeutic targets for the treatment of MCL and other B cell malignancies.

## TREATMENT OF MANTLE CELL LYMPHOMA

Because B-cell growth and response depends on the BCR signaling pathway, it is logical that B-cell malignancies such as Mantle-Cell Lymphoma are caused by deregulation of some component of this pathway. For this reason, many targeted therapies under current investigation and use inhibit genes involved in B cell receptor signaling. The following sections detail various agents that have demonstrated efficacy in the treatment of MCL through interaction with BCR pathway genes. However, it is important to note that even in the most efficacious of drugs, acquired resistance is common. Mutations could affect and constitutively activate any level of a pathway. This raises the possibility of cancer cells withstanding therapeutic agents that target specific B-cell pathway components. Knowledge of the B-cell signaling pathways downstream from receptor activation is crucial to recognizing and overcoming the mechanism of drug resistance. Studying the gene expression profiles of MCL samples might provide insight into the complex interplay of genes in lymphoma, particularly in resistant cases [24]. Conversely, understanding the current successful targeted therapies for MCL illuminates the BCR pathway actors that are commonly implicated in deregulated cell expansion.

### Bruton's tyrosine kinase inhibitors

Ibrutinib was designed to inhibit Btk by selectively interacting with an ATP-binding site in the tyrosine kinase domain [7]. BTK inhibitor ibrutinib showed durable efficacy in relapsed MCL as a single agent; specifically, such cases demonstrated a 68% overall response rate and 47% overall survival at 24 months [25]. It is currently considered the drug of choice in the treatment of relapsed/refractory patients that have undergone at least one prior therapy. However, acquired and primary resistance to ibrutinib has caused relapse in over 50% of patients; the patients who relapse after ibrutinib treatment have exhibited poor clinical outcomes. Current therapies have not been effective in post-ibrutinib relapsed cases, indicating that resistance-causing mutations must be discovered so that mechanism-specific agents can be applied to overcome them [26].

Several works have reported gene expression analysis data from cohorts of MCL patients and identified gene mutations that are associated with MCL [24]. A gene that is mutated in a small portion of MCL patients and that affects the BCR pathway is C481S [27]. This gene mutation alters the binding site of BTK so that ibrutinib may only bind reversibly, causing secondary ibrutinib resistance [28]. This leads to chronic activation of downstream BTK and mTOR/Akt pathways [29]. As stated above, BTK is involved in the activation of PLC-γ2, which propagates the B cell activation signal to PKCβ in order to trigger the classical NF-κB pathway, resulting in proliferation mediated by CDK-4 [17]. The mTOR/Akt pathway inhibits apoptotic factors and also signals CDK-4 mediated proliferation.

Ibrutinib resistance related to BTK mutations therefore has the potential to be overcome by treatment with a CDK4 inhibitor. When CDK4-dependent proliferation is inhibited, reversible ibrutinib activity is sufficient to down regulate BTK pathways. However, BTK mutation is not a very common mechanism in MCL [26]. Additional studies have shown that the mammalian target of rapamycin (mTOR), activated by Akt, is often mutated in patients with primary ibrutinib resistance [30]. Ibrutinib resistance is an active area of research. The T316A mutation affects the protein's SH2 domain, while the C481 residue is within its kinase domain [31]. Deregulation of BCR signaling components such as BTK is not the only factor that may contribute to ibrutinib resistance; the upregulation of anti-apoptotic molecules such as Bcl-2 may also dampen the efficacy of ibrutinib [32]. Additionally, sustained PIK3-Akt signaling has been detected in MCL patients with primary ibrutinib resistance [27, 33]. There have also been promising results from trials of drugs that target other molecules in the BCR pathway and may be used in combination with ibrutinib and could potentially overcome emergent ibrutinib resistance.

In light of the frequency with which MCL patients acquire resistance to ibrutinib, the search has begun for alternative BTK inhibitors. In addition to problems with resistance, ibrutinib has been known to cause bleeding, hematological events such as neutropenia, and atrial fibrillation [25]. It has been hypothesized that such toxicities are associated with ibrutinib's activity on targets other than BTK, such as EGFR, FGR, FRK, HER2, HER4, ITK, JAK3, LCK, BLK and TEC [34]. Several BTK inhibitors that are potentially more selective and less toxic than ibrutinib are currently undergoing varying phases of clinical trials. Below I will describe three of the most promising competitors of ibrutinib.

BGB-3111 is a small molecule inhibitor of BTK designed by BeiGene that recently entered phase II of a clinical trial designed to compare its efficacy and safety with that of ibrutinib in patients with refractory or relapsed MCL. In preclinical animal studies, BGB-3111 demonstrated superior oral bioavailability, achieving higher exposure and more complete target inhibition in the tissues than ibrutinib [34]. Alternative BTK inhibitors to ibrutinib are significant clinical developments because while ibrutinib has demonstrated a relatively high response rate of 68% in relapsed/refractory MCL, adverse events such as bleeding, neutropenia, and atrial fibrillation have been reported. These events are thought to be associated with the off-target effects of ibrutinib (binding to targets other than BTK) [35]. Therefore, more selective inhibitors may be more tolerable to patients.

While the most extensive testing of BGB-3111 has been done in Waldenstrom's macroglobulinemia (WM)—in fact, a phase 3 trial has recently been initiated by BeiGene—the inhibitor has been reported to be safe, tolerable, and highly active in patients with a variety of relapsed/refractory B-cell malignancies, based on a phase 1 trial in 2015. In this trial, 4 out of 6 MCL patients responded to BGB-3111 within a median of 227 days of treatment and 1 person experienced stable disease [34]. This phase 1 trial is the extent of the published data from clinical trials performed on MCL patients, but in March of 2017 BeiGene announced in the media that the dosing of the first patient in a single-arm phase II study of BGB-3111 in relapsed/refractory MCL, which is taking place in China; according to the clinical trial database of the National Institutes of Health, the estimated primary completion date is November 2018. Also, in the past two years trials in other B cell malignancies have progressed. A phase 1 trial in chronic lymphocytic leukemia (CLL) and small lymphocytic lymphoma (SLL), led by John Seymour, PhD, Director of Cancer Medicine at Peter MacCallum Cancer Centre in Victoria, Australia, is ongoing in addition to the phase 3 head-to-head comparison of BGB-3111 against ibrutininb in WM.

ACP-196 (Acalabrutinib) is another relatively new BTK inhibitor currently in phase 1 and 2 clinical trials for several B cell malignancies, such as CLL and NHL. ACP-196 inhibits BTK in the same manner as ibrutinib: by covalently binding to cys481 [35]. In a recent phase 1 trial in CLL, ACP-196 demonstrated a 95% overall response rate [36]. Open label, phase II studies of ACP-196 in previously treated patients with MCL are in progress. Furthermore, it has been shown to be more potent than ibrutinib because it has a lower IC_50_ against BTK than ibrutinib and has quicker oral absorption and favorable plasma exposure. ACP-196, like BGB-3111 and Ono, has a higher IC_50_ for other targets than does ibrutinib [35]. Its potency and selectivity indicates that ACP-196 could be a potential way around the toxicity associated with ibrutinib because it exhibits less off-target inhibition. The results of acalabrutib in 124 relapsed and or refractory MCL patients will be presented at the upcoming American Hematology Society Annual Meeting in December 2017.

Ono, or GS-4059, is another inhibitor of BTK that blocks auto-phosphorylation at the Tyr223 position. Ono/GS-4059 has been evaluated in DLBCL and MCL cell lines in combination with other agents. Ono and phsophotidylinositol 3 kinase inhibitor idelalisib showed synergistic activity in inhibiting the growth of a subset of these cell lines [37]. In a phase 1 clinical trial, Ono demonstrated a 92% (11 of 12) overall response rate in patients with MCL [38]. This trial was performed on a CLL cohort and an NHL cohort, and long-term follow-up of 17 CLL patients revealed that toxicity continues to be manageable and no dose-reducing toxicity occurred in these 17 patients [38]. Similarly to the BGB-3111 and ACP-196, Ono has achieved response rates comparable or favorable to that of ibrutinib while exhibiting a promising safety profile due to its greater selectivity. However, published data on these alternative BTK inhibitors remains somewhat limited and studies are ongoing.

### PI3K inhibitors

Another way to treat MCL through regulation of the BCR pathway is the inhibition of the PI3K portion of the pathway. Overexpression of PI3K/AKT appears to contribute to the pathogenesis of various lymphoid malignancies, including indolent NHL, MCL, and CLL. Inhibiting PI3K results in apoptosis [39, 40]. Idelalisib was the first approved PI3K inhibitor, and similarly structured duvelisib is still in development. Indeed, the PI3K pathway is a popular target for B-cell malignancy treatment today. A novel small-molecule inhibitor still early on in development is TGR-1202. All three listed therapies inhibit the delta isoform of PI3K, although duvelisib targets the gamma isoform as well [41, 42].

While these PI3K inhibitors have been studied most extensively and with best results in chronic lymphocytic leukemia, many studies include subjects with B cell lymphoma. A 2014 study of idelalisib in the treatment of 40 MCL patients achieved an ORR of 40%. Overall, 33 of 39 evaluable patients (84.6%) (1 patient did not have a tumor assessment) had reduction in lymph node size [19]. Since then, other promising therapies such as ibrutinib have been approved, but PI3K inhibition remains an efficacious strategy that could be useful in combined therapies to treat ibrutinib-resistant MCL.

In an effort to combat the common relapse in MCL patients on ibrutinib, Zhang et al. at MD Anderson Cancer Center studied the effects of combined ibrutinib and duvelisib in a PDX mouse model. The data demonstrated that the PI3K/AKT/mTOR signaling pathway was activated in both primary and acquired ibrutinib-resistant MCL cell lines and PDX MCL cells, and duvelisib significantly inhibited tumor growth and prolonged survival of MCL-PDX mice [42]. While PI3K inhibitors are not the first line of action in the treatment of MCL, the PI3K-mediated portion of BCR signaling is an active area of study for B-cell malignancies in general.

## RELATED TARGETS IN THE TREATMENT OF MANTLE CELL LYMPHOMA

The therapies described above are often used in combination with agents that target molecules on the periphery of the BCR pathway.

### Venetoclax (ABT-199)

Venetoclax is the commercial name for the drug ABT-199, which is an inhibitor of the anti-apoptotic gene BCL-2, which is activated by Akt and catalyzes glycolysis, although BCL-2 family proteins can have pro-apoptotic effects as well [20]. In a recent Phase I trial, single-agent venetoclax performed well in refractory/relapsed MCL, with a 75% overall response rate (21 patients responded our of 28 patients with MCL) and 14 month median progression-free survival—similar results to findings with ibrutinib [43]. This first-in-human Phase I study examined the response to venetoclax in several varieties of relapsed/refractory NHL, and MCL demonstrated the highest likelihood and durability of response.

A Phase II trial of a combined ibrutinib/venetoclax therapy is currently underway. As it is early in the study, the number of patients enrolled is small, but initial results are promising. The treatment design is 4 weeks of ibrutinib induction at the accepted daily dose of 560 mg, followed by introduction of venetoclax with a 4-week ramp up to 400 mg daily. At full recruitment, the study is predicted to show improvement in CR rate after 4 months (primary endpoint) from a historic 9% for IB alone, to at least 30%. Out of 3 patients in the study who completed staging at 4 months, 2 had achieved complete remission. Safety results are acceptable, with no serious adverse events related to the study drugs [28]. Thus, concurrent inhibition of BTK and BCL-2 has the potential to be more efficacious than inhibiting one of these BCR pathway components at a time.

### Rituximab

+FDA-approved over a decade ago, in 2006, rituximab (Rituxan) is a CD20 antibody and is considered a front-line therapy for MCL [44]. As discussed above, CD 20 is a surface marker found on B cells that, when ligated, assists in B cell activation. CD20 monoconal antibodies such as Rituximab induce cytotoxicity of CD20+ B cells by promoting BCR clustering and a cytosolic calcium flux, signaling mitochondria to release apoptosis-inducing cytochrome C [45]. Interestingly, cytosolic calcium flux can also activate BCR-dependent cell survival pathways involving proteins such as the transcription factor NF-κB and the mitochondrial membrane-stabilizing protein Bcl-2 [11]. Rituximab is most commonly used in concert with other therapies. In young newly diagnosed patients, treatment often takes the form of high dose chemotherapy in combination with rituximab, followed by autologous stem cell transplant (ASCT). In older patients, rituximab has demonstrated efficacy as maintenance therapy after induction chemotherapy (often CHOP or CVP) [46]. Results from a recent international phase III trial indicate that rituximab maintenance may be useful in young patients after ASCT as well, with 4-year overall survival of 78% [47].

More recent studies aim to investigate the use of rituximab in combination with other targeted drugs as potential front-line treatment strategies. For example, a study published in 2015 suggested rituximab plus lenalidomide as an alternative to the more aggressive initial therapies listed above, which may not be optimal for some patients owing to certain comorbidities. The study achieved promising results: an ORR of 87% at 30 months [48]. This treatment does not directly regulate the BCR pathway, but a more recent trial investigated a combination of rituximab with the notable first-generation BTK inhibitor: ibrutinib. Ibrutinib is not approved in treatment-naïve patients, and is effective as a single agent in relapsed/refractory MCL. A phase II trial has offered preliminary data supporting its use with rituximab in relapsed or refractory mantle cell lymphoma. The hypothesis is that rituximab may be more effective in targeting MCL cells associated with redistribution lymphocytosis because of its CD20-specific activity [30]. A Phase III trial (NCT02427620) is warranted and in the process of enrolling patients to study the response of MCL to ibrutinib with rituximab after cytarabine chemotherapy.

### Other inhibitors of note

Recall that Syk is a vital agent in the initiation of B cell activation, first phosphorylating CD79a/b and later phosphorylating important molecules such as BTK and PLC-γ2, setting of many downsteam events. The most-studied inhibitor of Syk is R406 [13]. Fostomatinib is a prodrug of R406 and has been extensively studied in non-hodgkin's lymphoma (NHL), and while it demonstrated some therapeutic activity in MCL, it was more effective in other histologies and less effective than the agents discussed above [49]. Additionally, several inhibitors the mTOR have undergone clinical trial, such as temsirolimus and everolimus. However, an international phase III study published in 2016 demonstrated that temsirolimus had a lower response rate and higher toxicity than ibrutinib in relapsed or refractory MCL patients [50]. While these agents are not go-to therapies for MCL, there is the potential that their efficacy could be increased when used in combination with other agents.

## CONCLUSIONS

The BCR signaling pathway is at the root of many B cell malignancies. Understanding the details of the BCR pathway is the foundation of designing targeted treatments for diseases such as Mantle Cell Lymphoma. At this point, the BCR signaling pathway is well-understood and ubiquitous in the research of B cell malignancy treatments. Components of the BCR signaling pathway, such as BTK and PI3K/Akt are the subject of several effective targeted therapies for MCL. Overcoming drug resistance in MCL patients is strongly related to our understanding of the molecular development of their MCL. In ibrutinib-resistant patients with a BTK mutation, several pathways peripheral to the canonical BCR signaling pathway might be up-regulated, such as the anti-apoptotic BCL-2 pathway or the alternative NF-KB pathway. Therefore, drugs targeting proteins such as BCL-2 whose activity is modulated by the BCR signaling pathway have produced promising responses in MCL, and show much potential when used in combination with ibrutinib or another BTK inhibitor.

There are new agents for the treatment of MCL under study, many of which target components of the BCR pathway—for example, Akt and ERK inhibitors, and CD19 antibodies. Several of these drugs are efficacious in other B cell malignancies and are showing initial promise in *in vitro* studies in Mantle Cell Lymphoma. Furthermore, while the drugs discussed in earlier sections are proven single-agent treatments, many current studies focus on the potential of combination therapies. The challenge for investigators will be to integrate new and conventional therapies into combinations to maximize response rates and patient safety and overcome drug resistance. Clinical trials have suggested that initial therapy is probably the best time to incorporate novel agents [7]. These new combination therapies are likely to incorporate agents that target key players in the BCR pathway or at least molecules that indirectly affected by the BCR activation signal. For this reason, it is important to continue deepening our understanding of BCR signaling as it relates to MCL. Genetic analysis has revealed mutations and epigenetic events in the pathogenesis of MCL, and there is a lot of variation among cases. Uncovering how deregulation of the BCR pathway plays into the pathogenesis of MCL will inform future treatments and how they can be tailored to fit each patient's needs.
